# FIL SSF intraocular lens opacification after pars plana vitrectomy with gas tamponade for traumatic lens luxation and retinal detachment: a case report and literature review

**DOI:** 10.1186/s12886-023-03126-6

**Published:** 2023-09-25

**Authors:** Danilo Iannetta, S. Febbraro, N. Valsecchi, A. Moramarco, L. Fontana

**Affiliations:** 1 Ophthalmology Unit, Dipartimento di Scienze Mediche e Chirurgiche, Alma Mater Studiorum University of Bologna, Bologna, Italy; 2 IRCCS Azienda Ospedaliero-Universitaria di Bologna, Via Pelagio Palagi 9 Bologna, Postal code, 40138 Bologna, Italy

**Keywords:** FIL SSF intraocular lens, Scleral fixation, Secondary IOL implant IOL opacification, Pars plana vitrectomy, lens luxation

## Abstract

**Background:**

To report a case of sutureless scleral-fixated hydrophilic intraocular lens (FIL SSF IOL, Soleko, Italy) opacification following pars plana vitrectomy surgery using sulfur hexafluoride (SF6) for traumatic lens luxation associated with retinal detachment.

**Case presentation:**

A 77-year-old woman was referred to our emergency department after blunt trauma in her right eye. At the ophthalmic evaluation, visual acuity was hand movement, biomicroscopy showed pseudoexfoliation syndrome and a traumatic lens luxation in the vitreous chamber. The patient underwent pars plana vitrectomy, subluxated cataract explantation, and FIL SSF IOL implant. During surgery, an inferior retinal detachment was encountered, requiring 20% SF6 gas tamponade. No adverse events were encountered. One month postoperatively, visual acuity (BCVA) improved to 0,3 logMAR. At the 3-month follow-up, the patient presented with BCVA of 0,5 logMAR, and biomicroscopy showed a minimal IOL opacification. Six months postoperatively, BCVA decreased to 1.0 logMAR, and diffuse, IOL opacification was noted at slit lamp examination. The patient refused any other surgical intervention for IOL exchange.

**Conclusions:**

Although hydrophilic IOL opacification gas related is known, to the best of our knowledge, this is the first case reported in the literature of FIL SSF IOL opacification after pars plana vitrectomy with gas tamponade for retinal detachment.

## Background

Blunt ocular trauma can lead to many severe ocular complications, one of which is crystalline lens luxation [1].

Correction of the resulting aphakia still represents a challenge for surgeons, since the absence of capsular support makes “in-the-bag” or ciliary sulcus intraocular lens (IOL) implantation not feasible [2].

Several alternative solutions have been proposed to manage those circumstances, such as anterior chamber IOLs (AC-IOLs), iris-fixated IOLs (IF- IOLs), or scleral-fixated IOLs for the posterior chamber (SF-IOLs) [2].

Optimal management is not consensual, since each of these techniques provides both advantages and disadvantages [3].

Moreover, many IOL-related postoperative complications, like IOL opacification, IOL dislocation, and refractive alterations are still a concern in cataract surgery, despite the great evolutions in IOL designs and materials [3]. [4]

IOL opacification represents a potential indication for lens removal, occurring more frequently in hydrophilic acrylic lenses. The main risk factors associated with this complication are those procedures that involve the injection of intraocular air or gas [4].

The FIL SSF IOL is a relatively new intraocular lens, specifically designed for sutureless scleral fixation (SSF). It is a single-piece, foldable, hydrophilic acrylic IOL with a 25% H_2_O content and UV filter. The two T-shaped harpoons that project from the closed haptics can be implanted and fastened to the sclera, thus allowing the IOL to be suspended in the posterior chamber without the use of sutures [2].

Lens injection requires a 2,2-mm corneal incision. According to different surgical implantation variants that have been described, the scleral plugs can be placed underneath lamellar scleral flaps, within scleral pockets, or directly beneath the conjunctiva [5–10]. Overall, FIL SSF IOL has shown a good safety and efficacy profile [11].

Herein, we describe the first case of FIL SSF IOL opacification that occurred three months after lens implantation and simultaneous pars plana vitrectomy with gas tamponade for traumatic lens luxation and retinal detachment.

## Case presentation

A 77-year-old Caucasian woman accessed our Ophthalmic Emergency Unit at the S.Orsola-Malpighi University Hospital in Bologna (Italy) complaining of blurry vision in her right eye. She reported that she suffered a blunt trauma in the same eye the day before. She was under medical treatment for arterial hypertension, while her past ocular history was of pseudoexfoliation syndrome (PEX), defined as the deposition of extracellular fibrillar-granular proteic material produced by the eye on all structures bathed in aqueous humour in the anterior segment.

At the ophthalmic evaluation, visual acuity of the affected eye was hand motion, and intraocular pressure (IOP) measured by Goldmann applanation tonometry (Haag-Streit, Koeniz, Switzerland) was 21 mmHg. Ocular motility was normal. Slit lamp biomicroscopy of the anterior segment showed very mild corneal edema, deep anterior chamber, pigment loss from the pupil margin, and no visible crystalline lens in the posterior chamber.

Gonioscopy was performed with a Goldmann 3-mirror lens, showing an open angle, pigmentation in the inferior quadrant, and no signs of angle recession in any quadrant.

At dilated fundoscopy, luxated lens was found in the vitreous chamber, and no retinal breaks were appreciated.

**Fig. 1:**
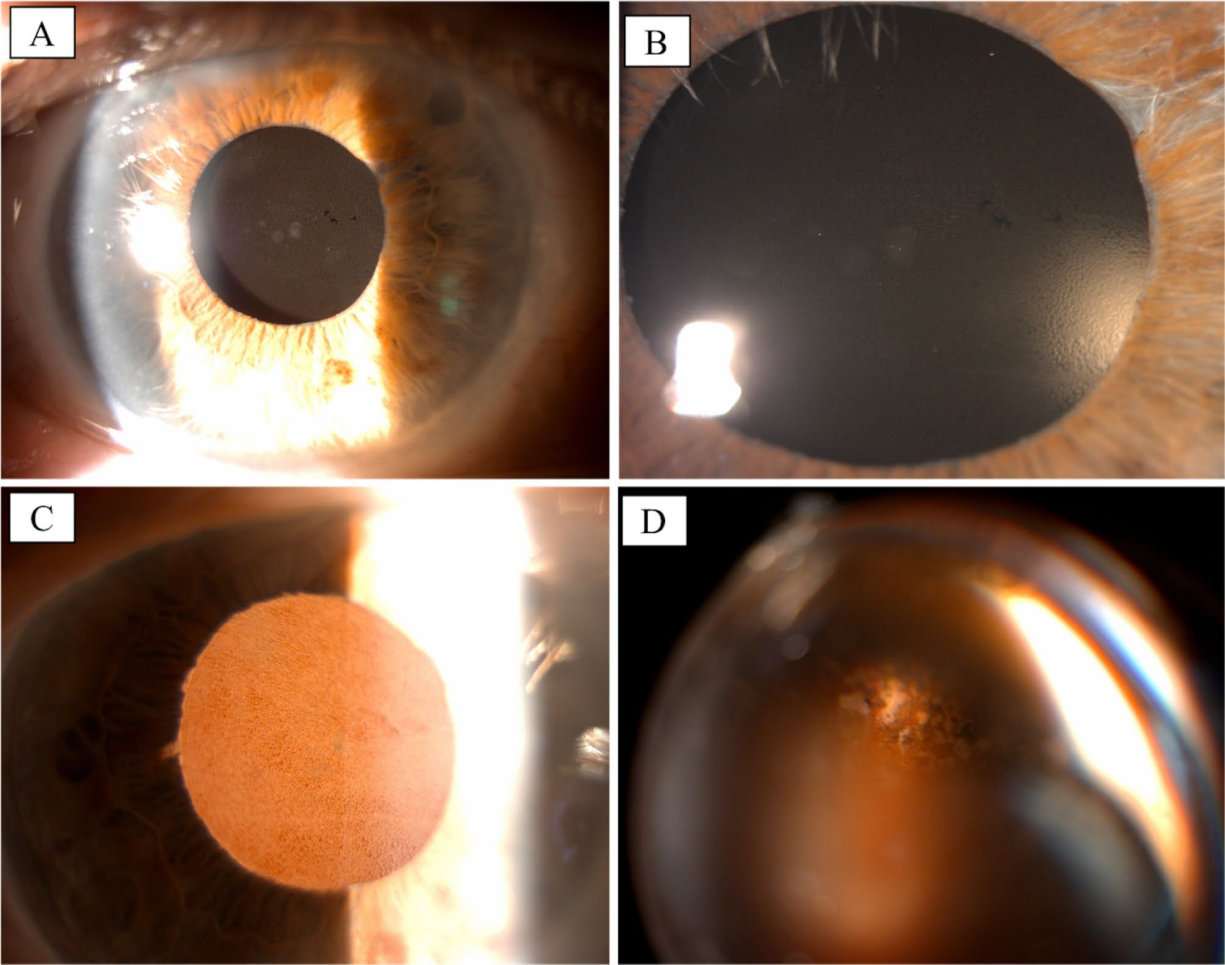
Clinical appearance of the opacified FIL SSF IOL 6 months after PPV with 20% SF6 injection. **(A)** and **(B)** Direct focal illumination. **(C)** Retro-illumination. **(D)** Fundoscopy was partially hindered by the IOL opacification. The laser treatment was visible in the peripheral inferior-temporal retinal quadrant

**Fig. 2:**
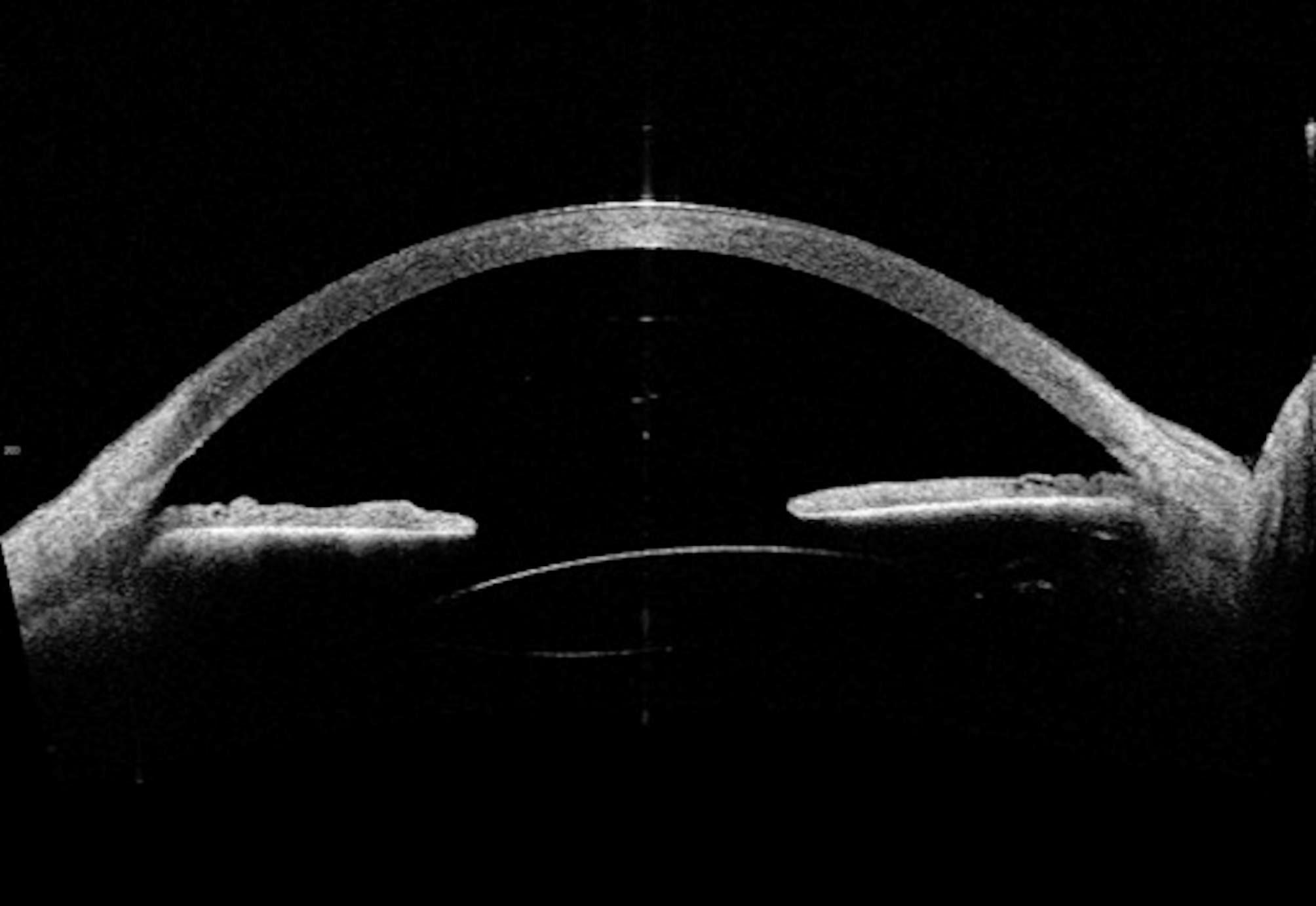
Anterior-segment Optical Coherence Tomography (AS-OCT) performed 6 months after surgery showed hyper-reflectivity of the anterior and posterior IOL surface

The day after, the patient underwent a 25-gauge pars plana vitrectomy (PPV) and lens explantation from the vitreous chamber under local anesthesia. During surgery, a small inferotemporal retinal detachment and a retinal break in the temporal retinal periphery were incidentally encountered. Once the FIL SSF IOL was implanted in the posterior chamber, according to the surgical technique previously described by Fiore et al. [9], the retinal break was laser-treated, and 20% sulfur hexafluoride (SF6) gas was injected into the posterior segment. No adverse events or complications were encountered during surgery. One month postoperatively, the best corrected visual acuity (BCVA) improved to 0,3 logMAR and IOP was 16 mmHg. At the slit lamp examination, the cornea was clear, and SSF-IOL was well-centered in the posterior chamber. Retina appeared well attached at the fundoscopy. However, at the 3-month follow-up visit, the patient presented with a visual acuity dropped to 0,5 logMAR, and rare central deposits were appreciated on the IOL surface during the slit lamp examination. Six months after surgery, the patient complained of severe visual impairment and glare. Visual acuity was 1 logMAR, and the slit lamp examination showed diffuse, dense IOL opacification with a granular pattern. See Fig. 1. Anterior segment Optical Coherence Tomography (AS-OCT) (CASIA 2, Tomey Corporation, Nagoya, Japan) was performed, showing mild hyperreflectivity of both the anterior and the posterior surface of the IOL. See Fig. 2.

At the last available follow-up at 10 months postoperatively, visual acuity and IOL opacification remained stable. The patient refused any other surgery for IOL explantation and exchange.

## Discussion and conclusions

IOL implantation and in-the-bag positioning currently represent the gold standard option in elective cataract surgery and secondary aphakia correction, since it offers excellent anatomic results and allows rapid visual recovery [2]. [10]

Unfortunately, there are many situations in which capsular bag support is insufficient or absent. These include ocular trauma with lens subluxation or dislocation in the vitreous cavity, metabolic or inherited conditions associated with zonular weakness (e.g., pseudoexfoliation, Marfan’s syndrome), and complicated cataract surgery [3].

To face such challenging situations, several alternative surgical approaches have been proposed. These include anterior chamber IOLs (AC-IOLs), iris-fixated IOLs (IF-IOLs), and scleral-fixated IOLs (SF-IOLs) [2].

Each of these solutions offers advantages and disadvantages. AC-IOLs and IF-IOLs implantations are technically easier but are associated with risks of corneal decompensation, iris chafing, and damage to the iridocorneal angle structures [12]. SF-IOLs are placed in the posterior segment, preventing contact with anterior segment structures, but have a higher risk of suprachoroidal or vitreous hemorrhage, retinal detachment, and lens decentration [13].

Surgical techniques for scleral fixation of IOLs have evolved over the years. Sutured scleral fixation techniques run the risk of suture erosion and associated risk of endophthalmitis, or lens dislocation due to broken suture material [8].

More recently, sutureless scleral fixation (SSF) techniques have been described, to reduce manipulation of ocular tissues and to avoid suture-related problems [14–16].

The introduction of the single-piece sutureless scleral fixation (SSF) lens (FIL SSF, Soleko, Italy) appeared as a remarkable step forward in managing these conditions. The peculiar shape of this IOL allows self-anchoring to the sclera through trans-scleral plugs, specifically designed for this purpose. Several published reports have shown encouraging anatomic and refractive outcomes of this solution. Self-centration, reduced surgical manipulation of ocular tissues, and preservation of the conjunctiva are among the advocated advantages, along with reduced surgical times and potential affordability for both anterior and posterior segment surgeons [2]. Nonetheless, various postoperative complications of cataract surgery might still occur. Opacification is one of them, and it can be defined as the loss of transparency of the IOL, and, though relatively rare, it can lead to impaired visual acuity, reduced contrast sensitivity, and glare, thus representing a potential indication for IOL removal or exchange [17].

In 2012, an observational multicenter retrospective study retrieving data from 15 different ophthalmological centers showed that IOL opacification represented the third most frequent reason for IOL explantation (11,3%), following dislocation/decentration (56,3%) and incorrect lens power (12,8%) [18].

In a recent retrospective cross-sectional study, Neuhann et al. found that IOL opacification was the most common reason for IOL removal (76,5%), while IOL dislocation was in the second rank (13,5%) [19].

In the attempt to optimize visual and anatomic outcomes, multiple IOL materials and designs have been introduced, such as hydrophilic acrylic, hydrophobic acrylic, and silicone [20]. Nevertheless, opacification has been observed with all of them. Many factors seem to play a role in the etiopathogenesis of opacification, such as IOL material, ocular and systemic diseases, and other surgical procedures performed [19].

According to timing, IOL opacifications can be divided into intraoperative/early postoperative (acute), if they occur in the first month after surgery, and late postoperative if the time interval between IOL implantation and opacification is longer [21].

Among causes that have been hypothesized for acute IOL opacification are: consolidation of water vapor and/or change in water content due to temperature fluctuation (causing the so-called IOL clouding), crystallization on IOL surface, secondary to the reaction between Calcium ions of irrigating solutions and phosphate ions of viscosurgical devices, discoloration secondary to intracameral dye, coating by ointments, and postoperative inflammation [21].

In a recent narrative review, Grzybowski et al. discussed two common types of late IOL opacification: glistenings and calcifications [22]. Glistenings are described as small fluid-filled vacuoles that form within the IOL polymer network, causing light scattering due to differences between the refractive indices of the vacuoles themselves and the IOL material [23]. This condition is mainly observed in hydrophobic acrylic IOL, and the pathogenesis likely involves temperature and osmotic changes that occur after lens implantation [4]. On the other hand, calcifications consist of deposits of calcium phosphate on the lens surface or subsurface and are predominantly observed in hydrophilic acrylic lenses. The formation of such deposits is deemed to be a multifactorial process. In 2008, Neuhann et al. suggested a classification of calcifications into three types [24]. Type 1 calcifications are those related to IOL itself (characteristics of polymer, packaging issues, etc.). Type 2 calcifications are those in which environmental factors likely played a crucial role. Type 3 calcifications include false positive diagnoses of IOL calcifications (“pesudocalcifications”). In a systematic review carried out in 2019, Fernàndez et al. found that different ocular and systemic diseases, such as diabetes mellitus (DM), arterial hypertension, and glaucoma, represent frequently reported conditions associated with IOL opacification [25]. Blood-aqueous barrier dysfunction and proinflammatory condition seem to be causative of calcifications in the case of DM. Other authors reported silicone IOLs calcifications coexisting with asteroid hyalosis. Asteroid bodies contain calcium and phosphate, and the process underlying their formation could be the same which leads to IOL calcification [26, 27].

Several surgical factors have been reported as potentially causative of IOL opacification. Among procedures appearing at most risk are those in which intraocular injection of air or gas is performed, such as Descemet Stripping with Automated Endothelial Keratoplasty (DSAEK/DSEK) and Pars Plana Vitrectomy (PPV) [25].

In a case series, Marcovich et al. reported 11 cases of hydrophilic acrylic IOL opacification after PPV involving intravitreal gas (e.g., SF6) injection. The complication was recorded 1 month – 6 years after PPV.

The authors hypothesized that direct contact between air/gas and the IOL surface may have led to dehydration and secondary local damage of the exposed area of the lens, thus creating a favorable substrate for calcium phosphate deposition from the aqueous humor [28].

Despite opacification can represent a complication for all IOL materials employed nowadays [4], it has been more frequently reported for hydrophilic acrylic lenses [25].

According to the aforementioned studies, we believe that, in our patient, the hydrophilic acrylic material of the lens and the intraocular injection of gas performed simultaneously with IOL implantation have likely played a crucial role in the genesis of calcium phosphate deposits on the IOL surfaces, thus leading to loss of lens transparency.

Coco et al. previously reported a case of Carlevale IOL opacification following Descemet stripping automated endothelial keratoplasty (DSAEK), where multiple re-bubblings with air were needed for graft detachments [29].

Two cases of transient intraoperative Carlevale IOL clouding were described, both of which ended up with a spontaneous resolution [30, 31].

Among other solutions for secondary IOL implantation, trans-scleral 4-point fixation of posterior chamber IOL – such as the Akreos AO60 (Bausch and Lomb, Bridgewater, NJ), a foldable hydrophilic acrylic IOL - using Gore-Tex sutures has shown good safety profile and clinical outcomes [32]. Both the Akreos IOL and the Gore-Tex sutures are used off-label for secondary implants. Localized or diffuse opacification of Akreos AO60 IOL was found to be a possible postoperative complication affecting visual acuity in various reports, with an incidence ranging up to 42% [33–38].

Retinal or corneal procedures involving the use of intraocular air or gas were deemed to be relevant risk factors [34].

In their case series, Patel et al. found that opacification occurred weeks after retinal detachment repair, regardless of the tamponade agent used, silicone oil or gas [33].

Transient lens opacification was also noted, probably related to postoperative intraocular inflammation [38].

In conclusion, this is, to the best of our knowledge, the first case reported in the literature of FIL SSF-IOL opacification, likely due to calcification, that occurred about three months after PPV with intraocular injection of gas.

Loss of lens transparency can affect visual acuity and sometimes represents a possible indication for IOL removal/exchange. FIL SSF IOL implant seems to represent a viable option in the management of insufficient or absent capsular support. Nevertheless, when other surgical procedures involving intraocular injection of air/gas are foreseen, the use of a hydrophobic IOL for secondary implantation should be contemplated as a preferential option.

## Data Availability

The data that support the findings of this study are available on request from the corresponding author.
